# Higher serum sCD14 is an independent biomarker for porto-sinusoidal vascular disease risk in CVID

**DOI:** 10.70962/jhi.20260066

**Published:** 2026-10-16

**Authors:** Laure Delaval, Karim Dorgham, Jehane Fadlallah, Emilie Corvilain, Pierre-Emmanuel Rautou, Eric Oksenhendler, Claire Fieschi, Guy Gorochov, Marion Malphettes

**Affiliations:** 1 Sorbonne Université, Inserm, Centre D’Immunologie et des Maladies Infectieuses, Paris, France; 2 National Reference Center for Adult Primary Immunodefiencies, CEREDIH, Hôpital Saint-Louis, Université Paris-Cité, Paris, France; 3 Department of Clinical Immunopathology, Assistance Publique – Hôpitaux de Paris, Hôpital Saint-Louis, Paris, France; 4 Université Paris-Cité, Inserm, Centre de Recherche sur L’inflammation, UMR 1149, Paris, France; 5 DMU DIGEST, Department of Hepatology, Assistance Publique – Hôpitaux de Paris, Hôpital Beaujon, Centre de Référence des Maladies Vasculaires Du Foie, FILFOIE, ERN RARE-LIVER, Clichy, France; 6 Assistance Publique – Hôpitaux de Paris, Groupement Hospitalier Pitié-Salpêtrière, Department of Immunology, Paris, France

## Abstract

Serum soluble CD14 (sCD14) is used as a surrogate marker for bacterial translocation. This cohort study demonstrates that higher baseline sCD14 levels are an independent biomarker for porto-sinusoidal vascular disease risk in patients with common variable immunodeficiency.

## Teaser

Delaval et al. demonstrate in this study that higher serum soluble CD14, a surrogate marker for bacterial translocation, and IgA deficiency are independent and early predictive biomarkers for porto-sinusoidal vascular disease risk in patients with common variable immunodeficiency.

## Introduction

Porto-sinusoidal vascular disorder (PSVD) is the main liver complication in patients with common variable immunodeficiency (CVID) and is associated with high morbimortality. We recently showed that gastrointestinal (GI) complications, together with low naive CD4^+^ T cell count, low serum IgA level, and lymphoproliferation, were strongly associated with the subsequent occurrence of PSVD in CVID patients (1). In line with this observation, PSVD was found to be associated with increased intestinal permeability in a study conducted mostly in non-immunocompromised patients, suggesting that bacterial factors derived from the gut may play a role in the pathogenesis of porto-sinusoidal endothelial alteration (2). Additionally, circulating bacterial 16S ribosomal DNA originating primarily from gut commensals can be detected in the serum of CVID patients and has been shown to be correlated with serum soluble CD14 (sCD14) (3). Here, we aimed to study the link between serum sCD14 levels, a surrogate marker for bacterial translocation, and PSVD.

## Patients and methods

This retrospective monocentric study was conducted within the prospective DEFI cohort dedicated to adult patients with predominantly antibody deficiency (1). Patients with CVID, as previously defined (1), could be enrolled as long as they did not have ongoing infection. Clinical data were retrospectively collected at that point, referred to as baseline. The time of last evaluation corresponds to the most recent information available as of February 2026. PSVD diagnosis was based on vascular liver disease group (VALDIG) criteria as stated in the Baveno VII consensus in patients evaluated every 6 mo with particular emphasis on screening for liver function tests, thrombocytopenia, and splenomegaly (1). Patients diagnosed with PSVD at inclusion and those who developed PSVD during follow-up were assigned to the PSVDincl and PSVDfu groups, respectively, both of which made up the PSVD+ group, while the PSVD− group comprised patients who had not developed PSVD at last evaluation. GI disorders were defined by the occurrence of infectious or non-infectious complications involving the GI tract, including atrophic gastritis, duodenal villous atrophy, inflammatory bowel disease, and lymphoid hyperplasia. At enrolment, baseline immunoglobulin level and immunophenotype were centrally determined while serum samples were collected and stored at −80°C until analysis. Serum sCD14 was quantified by ELISA (Quantikine ELISA kit, R&D). All samples were analyzed in duplicate. The DEFI cohort was approved by the appropriate institutional review board and ethics committee, and all patients provided written informed consent for participation and biological sample collection.

Continuous variables were expressed as median with interquartile range (IQR), and categorical variables as absolute numbers and percentages. Comparisons were performed using the Mann–Whitney U test for continuous variables and the χ^2^ or Fisher’s exact test for categorical variables. Cumulative incidences of PSVD were estimated considering death before PSVD diagnosis as a competing event, compared with Gray’s test and adjusted with a Fine and Gray model, whose stability was confirmed by bootstrapping (4). This analysis was performed in patients without PSVD at inclusion and with at least 1 year of follow-up, using a 1-year landmark. Predictive value of sCD14 level and other continuous covariates were assessed by integrated area under the curve (iAUC) derived from receiver operating characteristic (ROC) curves for censored event times with competing risks. iAUCs were calculated with time varying from 10 to 15 years. Optimal cut points were determined as cut points maximizing the Youden index (*J* = max_*c*_ {Se (*c*) + Sp (*c*) −1}) at the 12.5-year time point. *J* is Youden's index, *Se(c)* is the sensitivity at cut-off value *c,Sp(c)* is the specificity at cut-off value *c*, and max*__c__* denotes the maximum value obtained across all candidate cut-off values* c. *The covariates retained for multivariate adjustment were those we recently described as associated with the occurrence of PSVD in patients with CVID, namely baseline GI complications, lymphoproliferation, naive CD4^+^ T cell count, and serum IgA level (1). All statistical analyses were conducted using Stata (version 18.0, stcrreg package) and R (4.5.2 version, timeROC package). A two-sided P value <0.05 was considered statistically significant.

## Results

### Patient characteristics

A total of 272 CVID patients followed in the department and enrolled in the study between 2004 and 2019 had available baseline serum samples. Baseline characteristics were as follows: median age, 44.0 years (range, 14.6–87.8); median serum IgG, IgA, and IgM level, 5.34 g/L (IQR, 3.72–7.64), 0.10 g/L (IQR, 0.06–0.54), and 0.25 g/L (IQR, 0.07–0.50), respectively; median naive CD4^+^ T cell count, 148 × 10^6^/L (IQR, 49–314). Baseline prevalence of GI complications, lymphoproliferation, and autoimmunity was 41.2%, 56.6%, and 34.6%, respectively.

20 patients presented with PSVD at inclusion (7.4%), and 43 developed PSVD during follow-up (15.8%), for a total of 63 PSVD+ patients (23.2%). All PSVD+ patients had biopsy-confirmed diagnosis and documented portal hypertension. Median actuarial follow-up after inclusion was 13.7 years (95% confidence interval, 12.8–14.5). During the first year following inclusion, six patients died, and 11 were lost to follow-up without having been diagnosed with PSVD and were excluded from further analyses. 46 of the 255 remaining patients died during follow-up: 10/20 (50.0%), 15/43 (34.9%), and 31/192 (16.1%) in the PSVDincl, PSVDfu, and PSVD− subsets, respectively. 17 of the 25 PSVD+ patients (68%) died from PSVD complications.

### Increased serum sCD14 level was associated with PSVD

**Figure 1.**
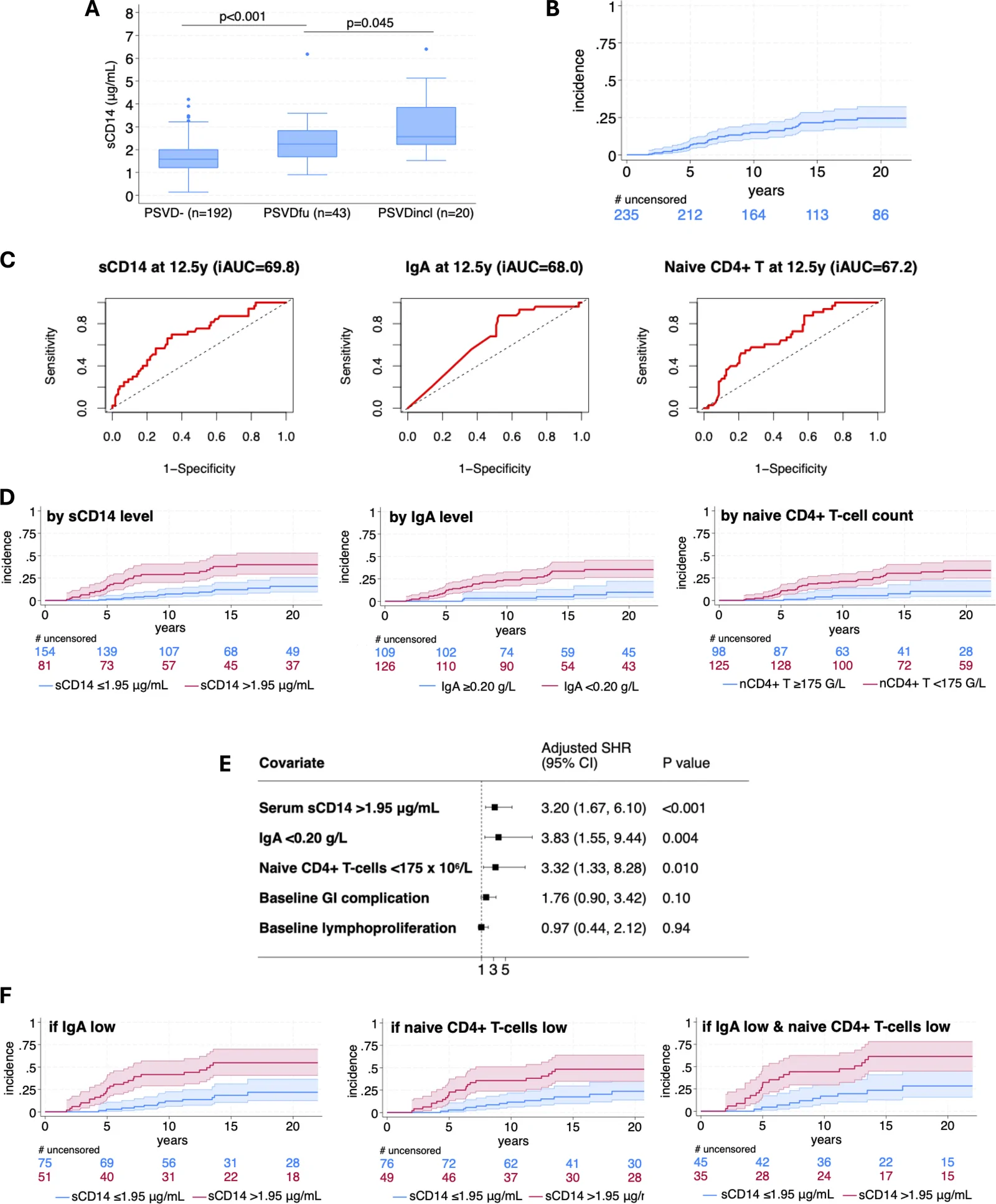
**Baseline serum sCD14 in CVID patients with or without PSVD. (A)** Median serum sCD14 levels in 255 CVID patients. PSVD−: patients without PSVD; PSVDfu: patients who developed PSVD during follow-up; PSVDincl: patients with PSVD at baseline. **(B)** Cumulative incidence of PSVD in 235 CVID patients without PSVD at baseline. **(C)** ROC curves for sCD14, IgA, and naive CD4^+^ T cells; iAUC (69.8, 68.0, and 67.2 for sCD14, IgA, and naive CD4^+^ T cells, respectively) were calculated with time varying from 10 to 15 years. Optimal cut points were then determined at the 12.5-year time point. Depicted curves are those obtained at the 12.5-year time point. **(D)** Cumulative incidence of PSVD in 235 CVID patients without PSVD at baseline according to baseline biomarker levels. At 12.5 years: (left) 32.4% (95% CI, 22.9–44.5) in patients with a baseline sCD14 level >1.95 µg/ml vs. 9.4% (95% CI, 5.3–16.4) in those with a lower level (SHR, 3.90 [95% CI, 2.09–7.26]; P < 0.001 by the Gray test); (middle) 27.2% (95% CI, 19.9–36.5) in patients with a baseline IgA level <0.20 g/L vs. 3.4% (95% CI, 1.1–10.1) in those with a higher level (SHR, 5.48 [95% CI, 2.35–12.79]; P < 0.001); (right) 25.5% (95% CI, 18.4–34.6) in patients with a baseline naive CD4^+^ T cell count <175 × 10^6^ cells/L vs. 5.3% (95% CI, 2.0–13.7) in those with a higher count (SHR, 4.42 [95% CI, 1.88–10.39]; P = 0.001). **(E)** Multivariate analysis for cumulative incidence of PSVD. Adjusted cause SHRs are indicated with 95% CIs for each covariables entering the Fine and Gray model. When compared with a Fine and Gray model omitting sCD14, SHRs of the four other covariates were very similar, and the addition of sCD14 substantially increased the overall Wald statistics of the model (not shown). **(F)** Added value of the sCD14 biomarker in predicting cumulative incidence of PSVD: At 12.5 years, (left) patients with baseline IgA level <0.20 g/L, 46.7% (95% CI, 33.5–62.0) in patients with a baseline sCD14 level >1.95 µg/ml vs. 13.7% (95% CI, 7.3–24.7) in those with a lower level (SHR, 4.01 [95% CI, 2.05–7.86]; P < 0.001); (middle) patients with baseline naive CD4^+^ T cells <175 × 10^6^/L: 40.6% (95% CI, 28.1–56.2) in patients with baseline sCD14 level >1.95 µg/ml vs. 15.2% (95% CI, 8.4–26.6) in those with a lower level (SHR, 3.10 [95% CI, 1.57–6.10]; P = 0.001); (right) patients with both baseline IgA <0.20 g/L and naive CD4^+^ T cells <175 × 10^6^/L: 50.8% (95% CI, 35.3–68.6) in patients with a baseline sCD14 level >1.95 µg/ml vs. 19.7% (95% CI, 10.3–35.6) in those with a lower level (SHR, 3.32 [95% CI, 1.58–6.95]; P = 0.001).

As shown in Fig. 1 A, baseline median serum sCD14 level was 2.57 μg/ml (IQR, 2.21–3.87), 2.24 μg/ml (IQR, 1.67–2.84), and 1.58 μg/ml (IQR, 1.20–2.01) in the PSVDincl, PSVDfu, and PSVD− patients, respectively. Overall, this level was higher in the 63 PSVD+ patients as compared with the 192 PSVD− patients (P < 0.001). Inter-subset comparisons are detailed in Fig. 1 A. Notably, median sCD14 level was significantly higher in patients with baseline GI complications (P = 0.003) while remaining low (median, 1.78 μg/ml) in the 12 patients with baseline granulomatous-lymphocytic interstitial lung disease (GLILD) without PSVD.

### Longitudinal serum sCD14 evaluation before and after PSVD onset

In 15 patients who developed PSVD during follow-up, a second sample was analyzed after PSVD diagnosis, showing a significant increase in median serum sCD14 levels: 2.43 µg/ml (IQR: 2.18–3.52) vs. 1.72 µg/ml (IQR: 1.47–2.76), P = 0.036.

### Increased serum sCD14 level was predictive of PSVD

In the 43 PSVDfu patients, the median time to develop PSVD was 6.5 years (IQR, 4.7–11.2). Fig. 1 B shows the cumulative incidence of PSVD in the 235 patients without PSVD at baseline, which was estimated at 15.0% (95% CI, 10.7–20.6) at 10 years and 21.5% (95% CI, 16.1–28.3) at 15 years. When analyzed as a continuous variable, higher serum sCD14 level at baseline was highly associated with PSVD development during follow-up (cause-specific HR [SHR], 2.33 [95% CI, 1.71–3.17]; P < 0.001 by the Gray test). ROC curves for sCD14 level, IgA level, and naive CD4^+^ T cell count are shown in Fig. 1 C. Optimal cut points were estimated at 1.95 μg/ml (sensitivity, 66.2%; specificity, 68.3%; *J*, 0.35), 0.20 g/L (sensitivity, 87.8%; specificity, 47.5%; *J*, 0.35), and 175 × 10^6^ cells/L (sensitivity, 87.7%; specificity, 41.5%; *J*, 0.29) for sCD14, IgA, and naive CD4^+^ T cell count, respectively. Cumulative incidences of PSVD according to these cut points are shown in Fig. 1 D.

### Multivariate analysis

In multivariable analysis, baseline sCD14 level above 1.95 μg/ml, IgA level <0.20 g/l and naive CD4^+^ T cells <175 × 10^6^/L were independently associated with the occurrence of PSVD, while baseline GI complications or lymphoproliferation were not (Fig. 1 E). A significant value of adding sCD14 to both IgA and naive CD4^+^ T cell markers in predicting PSVD occurrence was observed as compared with a model without sCD14 (detailed in Fig. 1 E legend) and illustrated in Fig. 1 F.

## Discussion

In this large cohort of CVID patients, we show that increased serum sCD14 level is strongly associated with PSVD. We observed a significantly higher serum sCD14 level in the 20 patients with PSVDincl. This is in accordance with a recent study reporting increased intestinal permeability and bacterial translocation in 17 PSVD patients (including 16 immunocompetent patients), using LPS and zonulin as bacterial translocation markers, which were also elevated in the absence of portal hypertension (2).

Furthermore, increased serum sCD14 level turned out to be an independent predictor of PSVD development. The median time to develop PSVD from baseline sCD14 assessment was 6.5 years, making serum sCD14 an early predictive factor. Importantly, this would also suggest that the process leading to PSVD may take years until portal hypertension is diagnosed.

The multivariate analysis provided more information compared with our previous observations (1). We confirmed that IgA deficiency remained independently correlated with PSVD development. While sCD14 and GI disorders were significantly associated at baseline, only sCD14 remained independently predictive of PSVD. This would suggest that digestive bacterial translocation may occur in the absence of obvious GI symptoms. Severe IgA deficiency could contribute to impaired gut microbiota, leading to mucosal barrier defects and commensal bacterial transcytosis, as recently shown (5). A weakness of this study is the lack of specificity of sCD14, an indirect marker of gut microbial translocation, released after monocyte/macrophage exposure to various microbial products. As a matter of fact, we found significantly increased serum sCD14 levels in patients with GI complications or PSVD, but not in the 12 patients with GLILD, another manifestation of systemic immune activation.

Given the correlation between sCD14 and circulating bacterial 16S rDNA, and given that gut-derived products are directly delivered to the liver, the present study provides additional evidence for the involvement of gut alteration, bacterial translocation, and subsequent immune activation in a context of IgA deficiency and T cell defect as a possible mechanistic explanation for the development of PSVD.

## Data Availability

The data underlying this study are not publicly available due to legal reasons: Individual patient data sharing is controlled by French laws. The data are available from the corresponding author upon reasonable request by further contracting.
